# Targeting cellular metabolism using rapamycin and/or doxycycline enhances anti-tumour effects in human glioma cells

**DOI:** 10.1186/s12935-018-0710-0

**Published:** 2018-12-19

**Authors:** Gábor Petővári, Zoltán Hujber, Ildikó Krencz, Titanilla Dankó, Noémi Nagy, Fanni Tóth, Regina Raffay, Katalin Mészáros, Hajnalka Rajnai, Enikő Vetlényi, Krisztina Takács-Vellai, András Jeney, Anna Sebestyén

**Affiliations:** 1 1st Department of Pathology and Experimental Cancer Research, Semmelweis University, Üllői út 26, Budapest, 1085 Hungary; 2 Hungarian Academy of Sciences-Momentum Hereditary Endocrine Tumours Research Group, Semmelweis University-National Bionics Program Budapest, Üllői út 26, Budapest, 1085 Hungary; 3 Department of Biological Anthropology, Eötvös Loránd University, Pázmány Péter sétány 1/A, Budapest, 1117 Hungary

**Keywords:** Glioblastoma, mTOR inhibitor, Anti-metabolic drug combinations, Tumour metabolism, Rapamycin, Doxycycline, Temozolomide

## Abstract

**Background:**

Glioma is the most common highly aggressive, primary adult brain tumour. Clinical data show that therapeutic approaches cannot reach the expectations in patients, thus gliomas are mainly incurable diseases. Tumour cells can adapt rapidly to alterations during therapeutic treatments related to their metabolic rewiring and profound heterogeneity in tissue environment. Renewed interests aim to develop effective treatments targeting angiogenesis, kinase activity and/or cellular metabolism. mTOR (mammalian target of rapamycin), whose hyper-activation is characteristic for many tumours, promotes metabolic alterations, macromolecule biosynthesis, cellular growth and survival. Unfortunately, mTOR inhibitors with their lower toxicity have not resulted in appreciable survival benefit. Analysing mTOR inhibitor sensitivity, other metabolism targeting treatments and their combinations could help to find potential agents and biomarkers for therapeutic development in glioma patients.

**Methods:**

In vitro proliferation assays, protein expression and metabolite concentration analyses were used to study the effects of mTOR inhibitors, other metabolic treatments and their combinations in glioma cell lines. Furthermore, mTOR activity and cellular metabolism related protein expression patterns were also investigated by immunohistochemistry in human biopsies. Temozolomide and/or rapamycin treatments altered the expressions of enzymes related to lipid synthesis, glycolysis and mitochondrial functions as consequences of metabolic adaptation; therefore, other anti-metabolic drugs (chloroquine, etomoxir, doxycycline) were combined in vitro.

**Results:**

Our results suggest that co-targeting metabolic pathways had tumour cell dependent additive/synergistic effects related to mTOR and metabolic protein expression patterns cell line dependently. Drug combinations, especially rapamycin + doxycycline may have promising anti-tumour effect in gliomas. Additionally, our immunohistochemistry results suggest that metabolic and mTOR activity alterations are not related to the recent glioma classification, and these protein expression profiles show individual differences in patients’ materials.

**Conclusions:**

Based on these, combinations of different new/old drugs targeting cellular metabolism could be promising to inhibit high adaptation capacity of tumour cells depending on their metabolic shifts. Relating to this, such a development of current therapy needs to find special biomarkers to characterise metabolic heterogeneity of gliomas.

## Background

Glioma is the most common highly aggressive, primary adult brain tumour. Based on the World Health Organization (WHO) Classification of Central Nervous System Tumours, both low grade and high grade gliomas can be divided into two categories according to the presence or absence of IDH1/2 mutations. This classification also has a prognostic significance—IDH-mutant gliomas confer significantly improved prognosis compared to IDH wild-type tumours. Testing for 1p/19q-codeletion status is required for further classification of low grade gliomas. As a consequence of an unbalanced translocation between chromosomes 1 and 19, whole-arm deletions of 1p and 19q arms are characteristic for oligodendroglial tumours. Other molecular alterations can also occur in gliomas such as EGFR amplification, p16/14 deletion, Notch overexpression, NF-1/PTEN co-deletion or other mutations e.g. EGFR, PDGFRA, PI3KCA, PTEN, CDKN2A/B and p53 [1, 2]. The recent treatments with surgical resection, followed by adjuvant radiation and chemotherapy—typically with temozolomide—result in 15-month median overall survival, with two-year relative survival rate in less than 30% of cases [3]. Other lower grade gliomas have better prognosis (median survival rate is approximately 5 years), however, recently gliomas have been considered as mainly incurable diseases. These led to continuous interest to develop more effective treatments targeting for example malignancy related angiogenesis [4], kinase activity and cellular metabolism [5, 6].

Tumour cells can adapt rapidly to alterations in tissue environment and therapeutic treatments depending on their metabolic shifts and profound heterogeneity [7]. To fuel energy-, macromolecule production and build new cells different metabolic rewiring processes are needed during tumour progression such as orchestrating TCA cycle, oxidative phosphorylation (OXPHOS), pentose-phosphate pathway, amino-acid and lipid synthesis etc. (DeBerardinis and Chandel summarise highlighted metabolic pathways implicated in tumour metabolism [7]). The consequence of oncogenic alterations influences the glycolytic-, glutaminolytic activity, mitochondrial OXPHOS, lipid metabolism and the expression/activity of related enzymes in cellular metabolic network. The expression and activity of the participating enzymes can mark metabolic alterations at cellular level. The glycolytic activity or mitochondrial OXPHOS capacity can be followed by the expression changes of hexokinase 2—HK2, phosphofructokinase—PFKP, lactate dehydrogenase A—LDHA, lactate dehydrogenase B—LDHB or β-F1-ATP synthase—β-F1-ATPase etc. Lipid metabolism is influenced by lipid synthesis and oxidation (can be characterised by the expression or activity of fatty acid synthase—FASN; acyl-coenzyme A synthetase short-chain family member 2—ACSS2; carnitine palmitoyltransferase 1A—CPT1a etc.). Glutamine consumption is characteristic for many tumours with increased glutaminase (GLS) expression [8, 9]. In parallel, intracellular metabolite concentrations (e.g. the main glycolytic product—lactate or other TCA metabolites—such as malate, pyruvate etc., the measured metabolites by the current study will included in a figure of the results section), several substrate transporter protein expressions (as glucose, glutamine and monocarboxylate transporters) and their activity could also be changed [10]. Based on the previously described metabolic enzyme expression alterations, recently there has been an increasing interest to target metabolism regulating pathways or metabolic enzymes in gliomas and other tumours [11].

mTOR kinase hyper-activation—which often occurs in different tumours—is linked mainly to PI3K/Akt signalling activation and alters cellular metabolism, protein turnover, cell growth and survival. mTOR kinase exists in two different complexes with characteristic proteins (such as Raptor and Rictor in mTORC1 and C2, respectively) and targets (S6K1, 4EBP1, SREBP1, ULK1, Akt etc.) [12]. mTORC1 controls anabolic processes such as protein synthesis and cell cycle entry in response to cellular energy, growth factor and nutrient availability. mTORC2—which is mainly influenced by growth factors—phosphorylates different downstream targets such as members of the AGC kinase family, Akt, serum/glucocorticoid regulated kinase (SGK) and protein kinase C (PKC) to promote lipogenesis, glucose uptake, glycolysis and cell survival [13].

The expression of several mTOR and metabolism related genes in normal human brain tissues has been studied previously at RNA and protein levels. According to immunohistochemistry studies and Protein Atlas expression database, ACSS2, ACSL1 (long-chain-fatty-acid-CoA ligase 1), CPT1a, GLS and additionally mTOR, Rictor expressions are low or undetectable (1+/0) in glial cells. Only S6 and Akt protein expressions were evaluated a slightly higher level (maximum 2+) in normal human glial cells. In other neuronal cells relatively higher mTOR, S6, Akt, ACSL1 and GLS expression levels have been detected.

Several phase I and phase II trials using mTOR inhibitors have been tested in mono-therapy and/or studied in standard chemo-radiation, however, these have not resulted in appreciable survival benefit [14, 15]. Combining anti-EGFR or anti-VEGF therapy with mTORI treatments could have certain significant benefits, however, these also have no real success. Recently it has also been described that the effects of rapalog (sirolimus) and temozolomide could be enhanced for example by chloroquine (an autophagy inhibitor) [16]. Despite these trials, GBM is still incurable which may be the consequence of glioma cell survival, metabolic plasticity and heterogeneity in developing glioma tissues [11, 14].

In our study, differences in mTOR inhibitor sensitivity in correlation to mTOR activity and other metabolic treatment combinations were studied using three human in vitro cultured glioma cell lines. Using mTOR inhibitors, temozolomide and other metabolic inhibitors (such as inhibitors of autophagy, beta-oxidation and mitochondrial functions) showed individual—cell line dependent—metabolic alterations in our mono-treatment study. Based on these, further two- or three-drug combinations were also tested; and the results suggest that co-targeting these different metabolic pathways—especially by rapamycin + doxycycline combinations—may have therapeutic benefits. In addition, for such targeting special biomarkers and the characterisation of metabolic heterogeneity of in vivo gliomas are needed as a part of personalised therapy, where side-effects have to also be considered.

## Materials and methods

All materials were purchased from Merck-Sigma-Aldrich (Darmstadt, Germany), except where it is indicated in the text.

### Cell cultures and different reagents

U251 (ECACC-09063001, characteristic glioma cell specific mutations in PTEN, NF-1, p53, MSH2), U87 (ATCC-HTB-14, characteristic glioma cell specific mutations in PTEN, NF-1 and Notch-2), U373 Uppsala (U373-U; ECACC-08061901) human glioma cells were cultured and treated in DMEM high glucose medium supplemented with 10% foetal bovine serum (FBS; HyClone), 2 mM L-glutamine and 100 UI/mL penicillin–streptomycin at 37 °C in a 5% CO_2_ atmosphere.

**Fig. 1:**
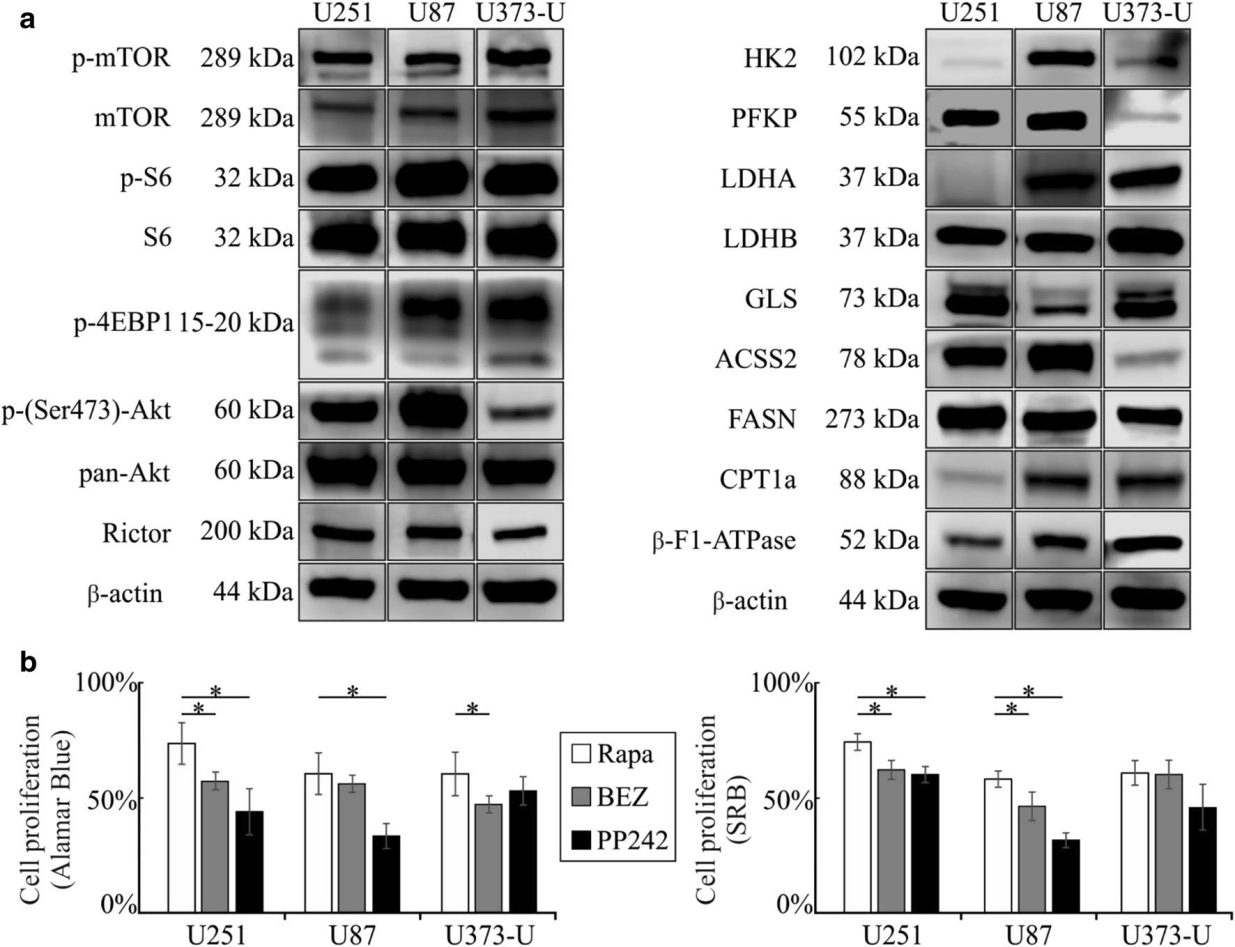
mTOR activity and metabolic differences in U251, U87 and U373-U human glioma cells. **a** mTOR activity related proteins and other metabolic enzyme expressions characterise and show some individual differences in the studied human glioma cell lines—representative figures of Western blot results; **b** the enzyme expression profiles could correlate to mTOR inhibitor sensitivity of glioma cells (rapamycin—Rapa 50 ng/mL; NVP-BEZ235—BEZ 1 µM; PP242 1 µM for 72-h treatments), which were monitored by Alamar Blue and SRB proliferation tests—the cell proliferation of untreated controls was considered 100%; rapamycin inhibited the proliferation in all studied cells, significantly; Significant differences compared to rapamycin were labelled by *p < 0.05

**Fig. 2:**
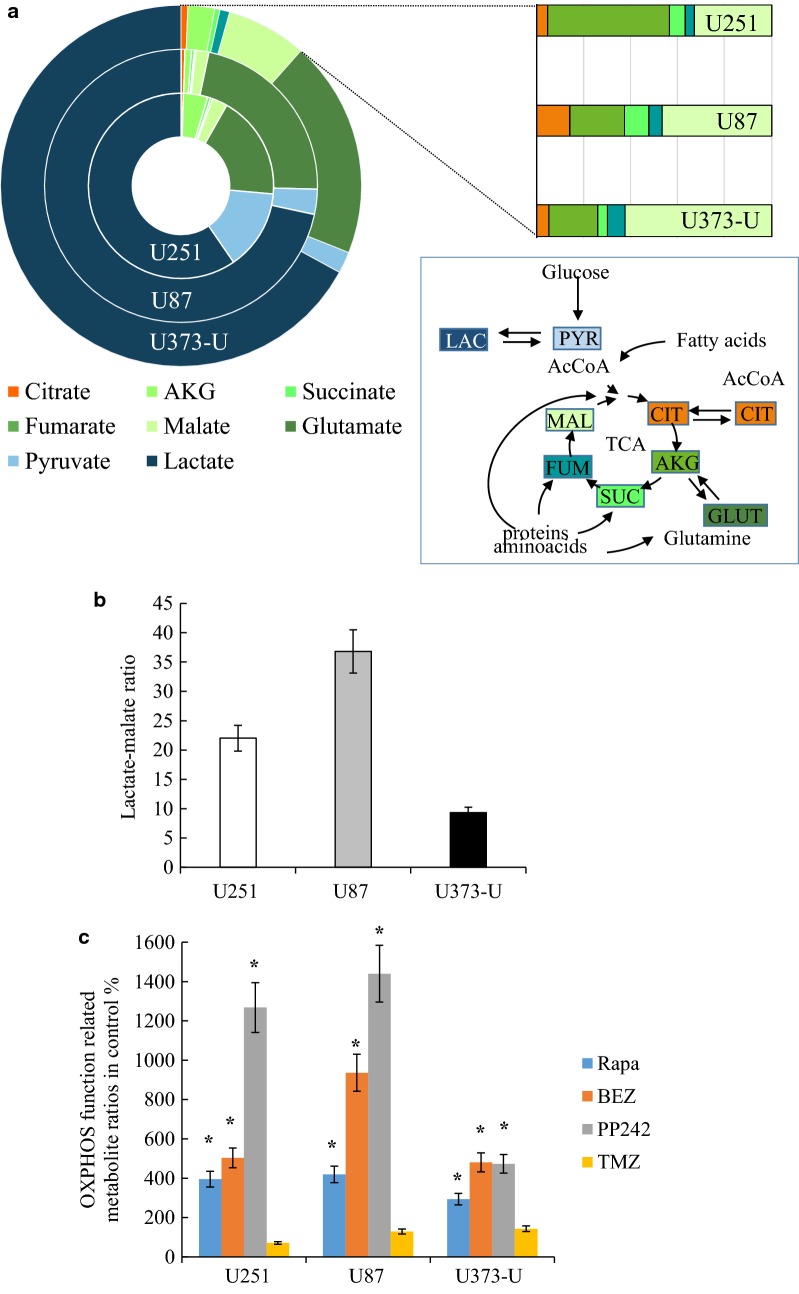
Intracellular metabolite concentrations in the studied three glioma cell lines. **a** High level of intracellular lactate or glutamate and differences in the concentration of other metabolites, such as pyruvate and several TCA cycle metabolites—metabolites were measured by LC–MS and the relative distributions of the detected *LAC* lactate, *PYR* pyruvate, *CIT* citrate, *AKG* α-ketoglutarate, *SUC* succinate, *FUM* fumarate, *MAL* malate, *GLUT* glutamate were given; **b** the highest lactate/malate ratio showed the highest glycolytic activity in U87 cells; **c** alterations in metabolite levels measured by LC–MS after 72-h mTORI and temozolomide treatments—(citrate/pyruvate)/(fumarate/succinate) ratios were given in untreated controls % to characterise OXPHOS function. Drugs were used in the following concentrations—rapamycin—Rapa 50 ng/mL; NVP-BEZ235—BEZ 1 µM; PP242 1 µM; temozolomide—TMZ 100 µM. Significant changes were labelled * (p < 0.05)

Using different assays cells were plated onto 96-well plates (2–5 × 10^3^ cells/well for proliferation tests) or into T25 flasks (3–6 × 10^5^ cells/flask—for LC–MS and Western blot experiments) and treated with different drugs and their combinations (rapamycin—Rapa, 50 ng/mL; NVP-BEZ235—BEZ, 1 μM, Cayman; PP242—1 μM, Tocris; doxycycline—Doxy, 10 μM; temozolomide—TMZ, 100 μM; etomoxir—Etom, 50 μM; and chloroquine—Chl, 50 μM) for 72 h, in combinations different drugs were added at the same time to the cell cultures. 1% DMSO was added to control cells and was used as a vehicle for some of the compounds as it was required. The concentration of the used mTOR inhibitors (Rapa, BEZ and PP242) was applied based on our previous publications [17, 18]. To study the effect of drug combinations lower dose than IC50 was applied in case of used metabolic or other inhibitors, these drug concentrations were defined by using others’ previous IC50 data and our preliminary experimental work in gliomas or other cancer types [19–22]. Alamar Blue (Thermo Fisher Scientific) and SRB tests were used to determine the anti-proliferative effects. At the end of different in vitro treatments a 4-h incubation period was performed with Alamar Blue. Fluorescence was measured in the 570–590 nm range by Ascent software (Fluoroskan Ascent FL fluorimeter; Labsystems International). In SRB assay, after 10% trichloroacetic acid fixation the cells were incubated with sulforhodamine B (15 min, 0.4 m/v%) following 10 mM Tris base addition. The absorbance was measured at 570 nm in a microplate reader. Percentage of the cell proliferation was given relative to control samples. At the beginning of experimental workflow both proliferation assays were performed and cell numbers were also followed after different treatments. No significant alterations were detected between the effects using different assays—these similarities were shown in Figs. 1 and 2.

To analyse the additive or synergistic effects of different drug combinations Combination Index (CI) was used [23]. The CI was calculated as $\frac{EA + EB}{EAB},$ where EA and EB were the individual effects of the mono-therapy and EAB was the observed combination effect. CI is within 0 and 1 means that the combined drugs have no additional effect on cells, in case CI is 1 indicates additive and above 1 synergistic effect, respectively.

### Metabolite analysis using liquid chromatography–mass spectrometry

Intracellular metabolites—lactate, pyruvate, citrate, α-ketoglutarate (AKG), succinate, fumarate, malate, glutamate, aspartate—were extracted based on Szoboszlai et al. with some modifications [24, 25]. Intermediates were extracted from cells and supernatants by methanol-chloroform-water (9:1:1) and vortexed at 4 °C. After centrifugation (15,000×*g*, 10 min, 4 °C) supernatants were stored at − 80 °C until liquid chromatography-mass spectrometry (LC–MS) measurements. The concentrations of lactate, pyruvate, citrate, AKG, succinate, fumarate, malate, glutamate and aspartate were assessed by using calibration curves obtained with the dilution of analytical grade standards in the range of 0.5–50 µM and the given ng/10^6^ cells concentrations were used in ratio calculation. LC–MS assays were used by Perkin-Elmer Flexar FX10 ultra-performance liquid chromatograph coupled with a Sciex 5500 QTRAP mass spectrometer. Chromatographic separation was carried out on a Phenomenex Luna Omega C18 column (100 × 2.1 mm, 1.6 µm) (GenLab Ltd., Budapest, Hungary). The mobile phase consisted of water and methanol containing 0.1% (v/v) formic acid. The MS was operating in negative electrospray ionisation mode. For the measurements the following settings were adjusted—source temperature: 300 °C ionisation voltage: − 4000 V, entrance potential: − 10 V, curtain gas: 35 psi, gas1: 35 psi, gas2: 35 psi, CAD gas: medium. Multiple reaction monitoring (MRM) mode was applied to perform quantitative analyses.

### Expression analysis of mTOR and metabolic proteins by Western blot

Protein extracts were lysed (50 mM Tris, 10% glycerol, 150 mM NaCl, 1% Nonidet-P40, 10 mM NaF, 1 mM phenylmethylsulfonyl fluoride, 0.5 mM NaVO_3_, pH 7.5) from at least 1 × 10^6^ cells and quantitated using Bradford protein reagent (BioRad) for Western blot analysis using sodium dodecyl sulfate polyacrylamide gel electrophoresis. After wet transfer PVDF membranes (BioRad) were incubated with the following antibodies: anti-phospho-mTOR (Ser2448) (p-mTOR; 1:1000; #2971, Cell Signaling Technology—CST), anti-mTOR (mTOR; 1:1000; #2972, CST), anti-phospho-S6 (Ser 235/236) (p-S6; 1:1000; #4858, CST), anti-S6 (S6; 1:1000; #2317, CST), anti-phospho-4E-binding protein 1 (Thr37/46) (p-4EBP1; 1:1000; #2855, CST), anti-Rictor (1:1000; #2140, CST), anti-phospho-Akt (p-(Ser 473)-Akt; 1:2000; #4060, CST), anti-Akt (pan) (pan-Akt; 1:1000; #4691, CST) anti-lactate dehydrogenase A (LDHA; 1:1000; #3582, CST), anti-lactate dehydrogenase B (LDHB; 1:2000; #85319, Abcam) anti-glutaminase (GLS; 1:1000; #15676, Abcam), anti-carnitine palmitoyltransferase 1A (CPT1a; 1:1000; #128568, Abcam), anti-fatty acid synthase (FASN; 1:1000; #3180, CST) anti-acyl-coenzyme A synthetase short-chain family member 2 (ACSS2; 1:1000; #3658, CST), anti-hexokinase 2 (HK2; 1:1000; #2867, CST), anti-phosphofructokinase (PFKP; 1:1000; #8164, CST) anti-β-F1-ATP synthase (β-F1-ATPase; 1:2000; #14730, Abcam) anti-pyruvate dehydrogenase (PDH; 1:1000; #3205, CST) and anti-β-actin (1:5000; #A2228, Sigma-Aldrich) were used as loading control. At the end biotinylated secondary antibodies, avidin-HRP complex (Vectastain Elite ABC Kit, Vector) and enhanced chemiluminescence technique (Pierce ECL Western Blotting Substrate using Li-Cor-C-Digit) were applied and the expression of proteins was quantitated by Image Studio Digits program.

### Patients, tissue samples and immunohistochemistry

Isocitrate dehydrogenase (IDH) wild-type (n = 10), IDH-mutant (n = 8) human glioblastoma and high-grade astrocytoma tissues were studied, two cases harboured 1p19q trisomy or tetrasomy. Samples were obtained by surgical resection at National Institute of Clinical Neurosciences (Budapest), between January 1, 2014 and December 31, 2015. Peritumoral brain tissue (n = 2) was also analysed as control. The archived FFPE tissue blocks were used with the approval of the Hungarian Scientific Council National Ethics Committee for Scientific Research (No. 7/2006). All cases were re-reviewed and re-classified based on The 2016 World Health Organization Classification of Tumors of the Central Nervous System [1].

Immunohistochemistry was performed on tissue microarrays (TMAs) with double scores per patients. Representative areas were selected by a neuropathologist for TMA construction. After deparaffinization, the tissue sections underwent antigen retrieval (citrate buffer pH 6, in a pressure cooker). Slides were incubated with the appropriate primary antibodies which were used in Western blot analyses to detect LDHA, GLS, CPT1a, FASN, ACSS2, HK2 or were established previously to detect p-mTOR activity related proteins (p-mTOR Ser2448—1:100; #2976, CST; p-S6 Ser 235/236—1:100; #2211, CST; Rictor -1:1000 A500-002A, Bethyl; phospho-Akt (p-(Ser473)-Akt—1:100; #4060, CST) at room temperature for 90 min, followed by Novolink Polymer Detection System (Novocastra, Wetzlar, Germany), DAB (Dako) chromogen and haematoxylin counterstaining. A scale of 0–3 was used for semiquantitative analysis of immunoreactivity by two independent pathologists (using Panoramic Viewer Software—3D Histech) as following: no staining, 0; weak staining, 1+; intermediate staining, 2+; and strong staining, 3+.

### Statistical analysis

The data are presented as mean ± SD deviation and calculated from three independent experiments with at least three or more parallels, depending on the used method. Data evaluation of in vitro experiments was performed using Student’s t (two-tailed) test and one-way analysis of variance (ANOVA). IBM SPSS software (version 22; SPSS Inc, Chicago, IL) was used to evaluate multiple comparisons. p < 0.05 was considered statistically significant.

## Results

### Differences in metabolic enzyme expressions and mTORI sensitivity in human glioma cells

The studied IDH wild-type glioma cell lines with different known oncogenic mutations (see in methods) showed high mTOR activity, due to well-known characteristics of PTEN loss and/or PI3K/Akt hyper-activation. High expression levels of proteins related to both mTORC1 and C2 activity were characteristic for the studied glioma cells, especially p-S6, p-4EBP1 and p-(Ser473)-Akt. U373-U showed relatively lower Rictor and p-(Ser473)-Akt expressions than the others (U251 and U87 cells). After 72-h mTORI treatments we could detect certain significant anti-proliferative effects of mTORC1 inhibitor (rapamycin) and other dual (NVP-BEZ235—BEZ) or ATP competitive (PP242) mTOR inhibitors using Alamar Blue and SRB proliferation tests. In correlation to the lower Rictor and p-(Ser473)-Akt expression, PP242 (mTORC1 and C2 inhibitor) had no significantly higher anti-proliferative effect than rapamycin in U373-U cells, as expected. These results highlighted the known potential importance of slight differences in mTOR activity and mTORI sensitivity in glioma cells (Fig. 1a, b).

Analyses of other bioenergetic pathway related enzyme expression profiles (Fig. 1a) and intracellular metabolite concentrations in these cells revealed further differences. U251 and U373-U cells showed lower glycolytic capacity related to low HK2 and in parallel, altered PFKP/LDH expression patterns. U87 cells expressed higher level of glycolytic enzymes than the other two cell lines. Individual GLS, ACSS2 and β-F1-ATPase expression levels were also detected. Moreover, FASN expression was relatively higher than CPT1a in all studied glioma cells which can be in correlation to the growing cell culture characteristics and metabolic shifts towards anabolic pathways (Fig. 1a).

Relating to the manifold potentially useful bioenergetic capacity, we measured high lactate, glutamate and detectable amount of TCA cycle metabolites in all studied cells by LC–MS. The intracellular lactate concentration, the lactate/malate ratio and its inverse correlation to pyruvate levels reflected to the protein expression profile of the related enzymes (HK2, LDHA, PKFP) (Figs. 1a, 2a). In our previous work the intracellular lactate/malate ratio was suggested as a potential metabolic marker [24, 25], these ratios showed the highest glycolytic activity in U87 cells and lower in U251 and U373-U cells (Fig. 2b).

### mTORI and temozolomide induce metabolic alterations in glioma cells

**Fig. 3:**
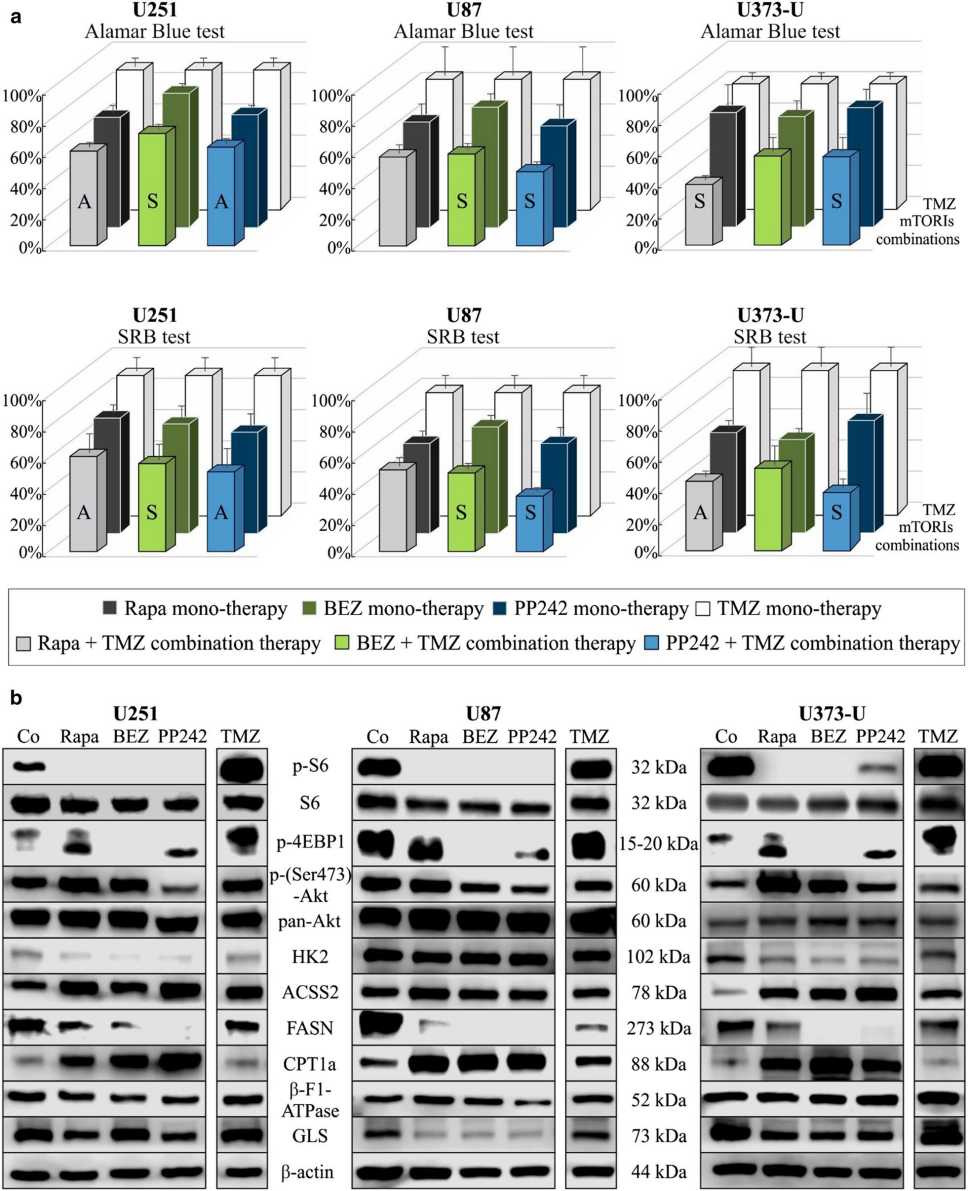
The anti-proliferative effects and alterations in mTOR activity and other metabolism related protein levels in response to mTORI and temozolomide treatments in human glioma cell lines. **a** mTOR inhibitors (rapamycin—Rapa 50 ng/mL; NVP-BEZ235—BEZ 1 µM; PP242 1 µM) and temozolomide (TMZ 100 µM) 72-h combination treatments effectively inhibited the proliferation of U251, U87 and U373-U cells—Alamar Blue and SRB proliferation test results, the cell proliferation of untreated controls were considered 100% (temozolomide slightly reduced the proliferation and had no significant growth inhibitory effect on U251, mTORIs had similar effects as in Fig. 1a, and all temozolomide + mTORI combined treatments had significant anti-proliferative effects, p < 0.05; the additive (A) or synergistic (S) effects of inhibitor combinations were given based on CI calculation, SD was added); **b** altered expressions of mTORC1 and C2 activity related proteins and other metabolic enzymes were also shown after different mono-treatments (results of representative Western blots after 72-h treatments)

It was suggested and described that mTORIs can sensitise gliomas to therapeutic temozolomide treatments. In this study, we tested the anti-proliferative effect of different mTORIs and temozolomide in combinations. Rapamycin was more effective than temozolomide in each cell line tested using Alamar Blue and SRB assays. Moreover, rapamycin + temozolomide combinations showed significantly enhanced anti-proliferative effects in the studied glioma cells as expected. Rapamycin + temozolomide combination was highly effective—synergistic—in U373-U glioma cells but this combination was less effective in the other two cells (U251, U87) (Fig. 3). We analysed the expressions of certain mTORC1 and C2 complex activity related proteins and other metabolic proteins after applying mTORIs or temozolomide. The effectivity of mTORIs was monitored by checking p-S6 and p-4EBP1 levels. These showed that all mTORIs reduced the phosphorylation of S6, however‚ in the studied cell lines mTORC1 and C2 inhibitors could only reduce the p-4EBP1 level. mTORIs resulted in downregulated HK2, GLS and lipid synthesis enzyme (FASN) expression and in parallel upregulated beta-oxidation enzyme (CPT1a) and ACSS2 levels in a cell line dependent manner (e.g. GLS expression was not decreased after BEZ treatment in U251; and HK2 expression was not altered in U87 cells).These data correlate with inhibited mTORC1 functions in parallel with the altered expressions of certain studied glycolysis, glutaminolysis and lipid metabolism related enzymes. The rapamycin-mediated upregulation of mTORC2 complex activity (p-(Ser473)-Akt increase) was shown in U251 and U373-U cells, and this was not observed (p-(Ser473)-Akt expression was lower) with dual PI3K-mTOR inhibitors (neither BEZ nor PP242) in all cells (Fig. 3).

Temozolomide significantly elevated p-4EBP1 levels in glioma cells and increased S6 phosphorylation in U251 but this was decreased in U87 and U373-U cells in contrast to the effects of mTORIs. In addition, temozolomide decreased lipid synthesis related FASN without influencing CPT1a expression in the studied glioma cells (Fig. 3).

In our previous metabolic studies of other tumour cells, the lactate/malate ratio was suggested using as a part of metabolic characterisation. In the current study, after 72-h different mTORI and temozolomide treatments we found that concentration changes of other metabolites need to be also considered to follow and analyse the metabolic changes at cellular level. Concentrations of other glycolytic and TCA cycle metabolites (e.g. pyruvate, citrate, succinate and fumarate) were considered to reveal the sign of glycolytic-OXPHOS alterations. According to our data, the ratios of citrate/pyruvate and fumarate/succinate showed the inhibited glycolytic and induced mitochondrial oxidation processes (OXPHOS shift)—significant changes were shown in Fig. 2c. The sign of such a significant alteration which is characteristic for OXPHOS shift was not detected in response to temozolomide treatments. These findings are in line with the fact that enzyme expression profile alterations are not characteristic for inhibited glycolysis and induced oxidation response in temozolomide treated glioma cells (Fig. 3b).

### The effects of doxycycline, etomoxir and chloroquine in human glioma cells

**Fig. 4:**
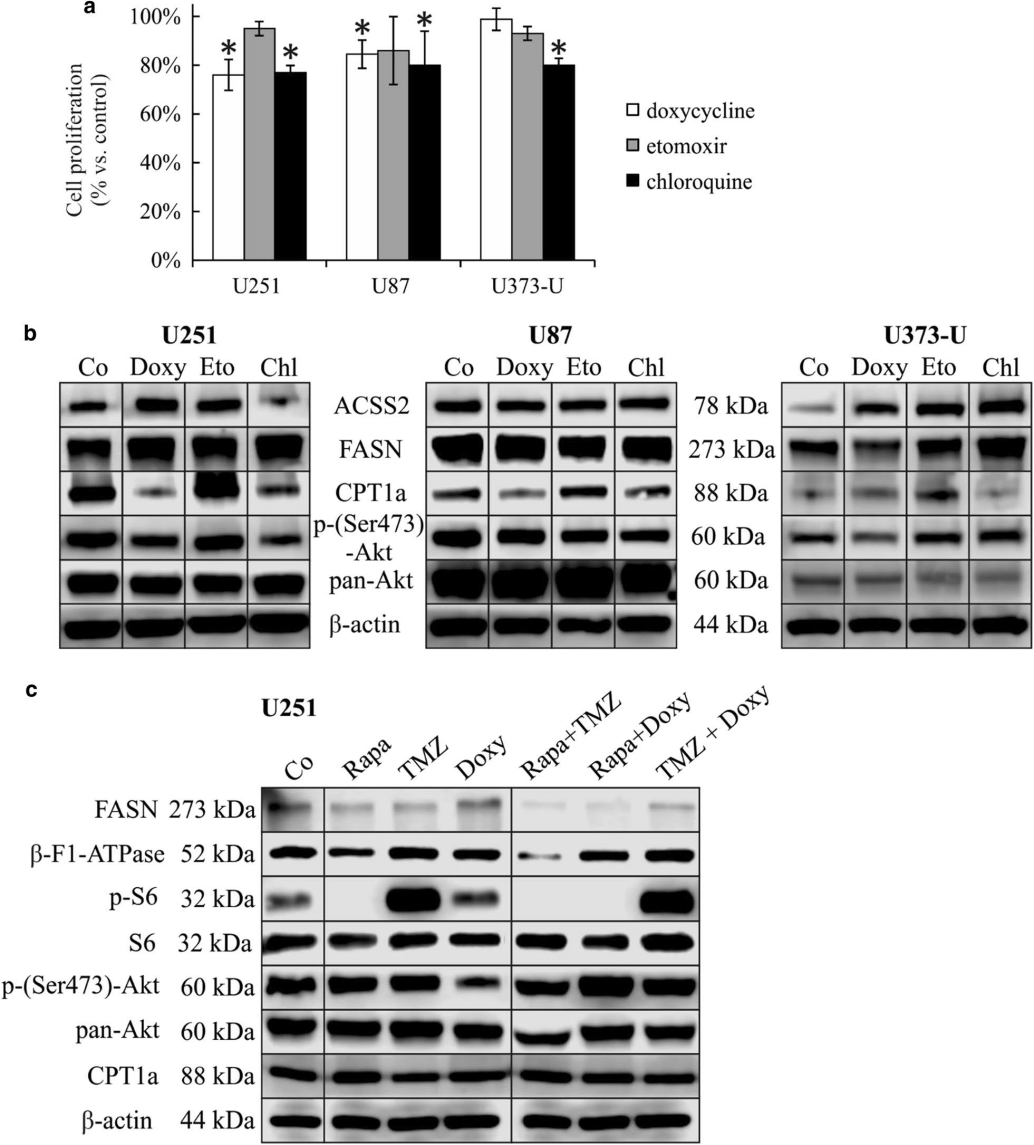
Metabolic drugs induced proliferative and protein expression profile changes in human glioma cells. **a** Doxycycline (Doxy 10 µM), etomoxir (Etom 50 µM), chloroquine (Chl 50 µM) 72-h treatments have slight growth inhibitory effects in U251, U87 and U373-U (Alamar Blue proliferation test data; *p < 0.05); **b** ACSS2, FASN, CPT1a and p-(Ser473)-Akt protein expressions were influenced by rapamycin, doxycycline, etomoxir and chloroquine after 72-h mono-treatments; **c** FASN, β-F1-ATPase, p-S6, p-(Ser473)-Akt and CPT1a protein expression compared to the untreated controls after applying two-drug combinations in U251 glioma cells (50 ng/mL rapamycin + 100 µM temozolomide—Rapa + TMZ; 50 ng/mL rapamycin + 10 µM doxycycline—Rapa + Doxy; 100 µM temozolomide + 10 µM doxycycline—TMZ + Doxy (Western blot results)—representative Western blot figures

Based on these alterations, other anti-metabolic treatments were also performed in our in vitro study; mitochondrial functions, lipid metabolism and autophagy were targeted. Slight anti-proliferative effects (10–25% reduction) of doxycycline (mitochondrial translation inhibitor using 10 µM [29]), etomoxir (beta-oxidation inhibitor using 50 µM) and chloroquine (autophagy inhibitor using 50 µM) were detected in almost all 72-h mono-treatments in vitro (Fig. 4a). In response to the above treatments we could observe the following individual differences in protein expression patterns: (a) in parallel with low anti-proliferative effects, doxycycline decreased p-(Ser473)-Akt level in all cells, significantly increased ACSS2 expression in U251 and U373-U; (b) etomoxir treatments increased CPT1a and ACSS2 expressions—these alterations could compensate metabolic stress in case of inhibited lipid oxidation. Although etomoxir did not alter significantly any other investigated protein levels, neither mTOR activity related p-S6/p-(Ser473)-Akt nor others; (c) The autophagy inhibitor chloroquine decreased CPT1a level and increased FASN expression significantly in U373-U and U251 cells. Decrease in p-(Ser473)-Akt and ACSS2 expression levels were also detected in U251 and U87 cells after chloroquine treatments (Fig. 4b).

### Effects of combined treatments

**Fig. 5:**
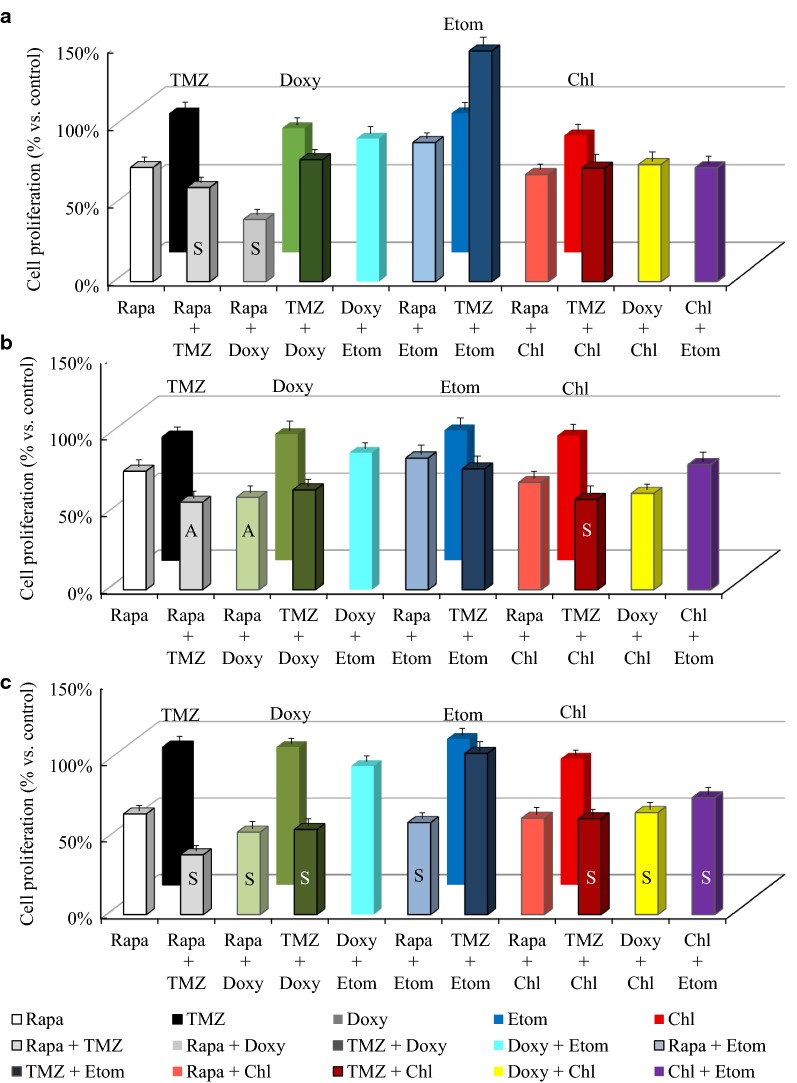
Rapamycin and temozolomide combined with other metabolic inhibitors have different in vitro anti-proliferative effects in human glioma cell lines. Alamar Blue test results after 72-h different two-drug combined in vitro treatments in U251 (**a**), U87 (**b**) and U373-U (**c**) glioma cells (rapamycin—Rapa 50 ng/mL, temozolomide—TMZ 100 µM, doxycycline—Doxy 10 µM; etomoxir—Etom 50 µM; chloroquine—Chl 50 µM). The additive (A) or synergistic (S) effects of combinations were given based on CI calculation, SD was added, the anti-proliferative effects were significant (p < 0.05) in almost all combined treatments compared to untreated cultures except for doxycycline + etomoxir and temozolomide + etomoxir treatments

As drugs like doxycycline, etomoxir and chloroquine exerted lower effects in mono-therapy, we decided to combine them further to target the metabolic adaptation in temozolomide and rapamycin treatments. Our findings suggest that certain of these new two-drug combinations—such as temozolomide and rapalogs—had rather cell line dependent additive effects (Fig. 5) relating to mTOR activity and other metabolic enzyme expression patterns. While etomoxir/chloroquine in combination with rapamycin or temozolomide were less effective two-drug treatments; temozolomide + chloroquine and rapamycin + chloroquine combinations were additive in U251 and U373-U cells, respectively. Doxycycline increased the effect of rapamycin in all glioma cells (the effects were synergistic in U251 and U373-U cells), moreover, in U87 cells this antibiotic treatment could induce the effect of rapamycin + temozolomide treatment (see below), as well (nevertheless rapamycin + doxycycline combination was not synergistic in U87 cells).

In U251 cells‚ we compared p-S6, p-(Ser473)-Akt, FASN, CPT1a and β-F1-ATPase expression changes further after using two-drug (rapamycin + temozolomide/doxycycline or temozolomide + doxycycline) combinations (Fig. 4c). Our results suggest that these drug combinations, especially rapamycin and doxycycline mutually affect the mechanism of each other’s metabolic adaptation. In U251 cells p-S6 and p-(Ser473)-Akt expressions were reduced (Fig. 4c) in response to rapamycin + doxycycline/temozolomide combinations. However, lipid metabolism related enzymes did not follow these expression changes in correlation to lowered mTOR activity (CPT1a and FASN expressions were not significantly changed). Moreover, β-F1-ATPase expression (as a result of altered—collapsed—mitochondrial OXPHOS) was downregulated following these—rapamycine + temozolomide/doxycycline—two-drug treatments (Fig. 4c).

**Fig. 6:**
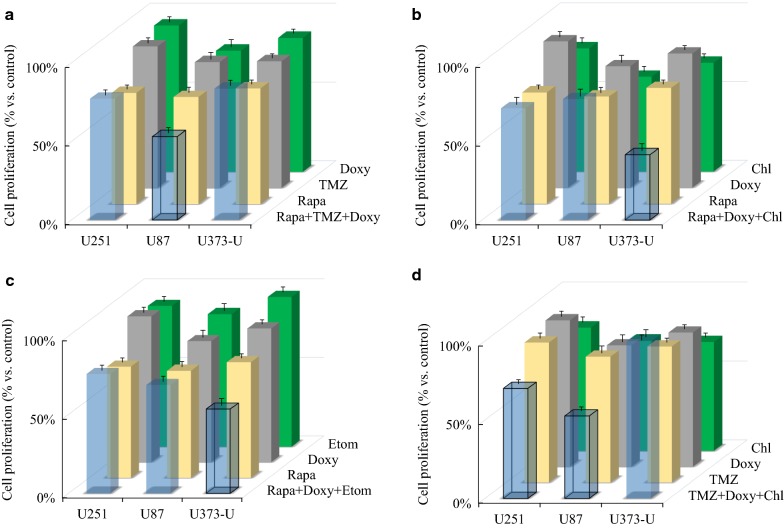
Certain interesting three-drug combination results in human glioma cells. Alamar Blue test results after 72-h different three-drug combinations in vitro treatments. The results of the most effective combinations, the significant effects of three drug combinations versus mono-treatments (p < 0.05) were indicated with framed columns, SD was added. Applied drug concentrations: rapamycin—Rapa 50 ng/mL, temozolomide—TMZ 100 µM, doxycycline—Doxy 10 µM; etomoxir—Etom 50 µM; chloroquine—Chl 50 µM in different combinations were the following: rapamycin + temozolomide + doxycycline—Rapa + TMZ + Doxy; rapamycin + doxycycline + chloroquine—Rapa + Doxy + Chl; rapamycin + doxycycline + etomoxir—Rapa + Doxy + Etom; temozolomide + doxycycline + chloroquine—TMZ + Doxy + Chl)

Furthermore, the effects of three-drug combinations exerted on proliferation were also tested in glioma cell lines. Based on our 72-h proliferation data, we found that the anti-proliferative effects of rapamycin + temozolomide treatments were significantly increased by adding doxycycline to U87 cells as it was described above. Rapamycin + temozolomide + doxycycline was effective, but could not induce the effect of either mono-therapy in U251 or U373-U. Other three-drug combinations also seemed to be very effective such as rapamycin + doxycycline + chloroquine/etomoxir in U373-U cells or temozolomide + doxycycline + chloroquine in U251 and U87 (Fig. 6).

### Heterogeneity of metabolic protein expression in human glioma biopsies

**Fig. 7:**
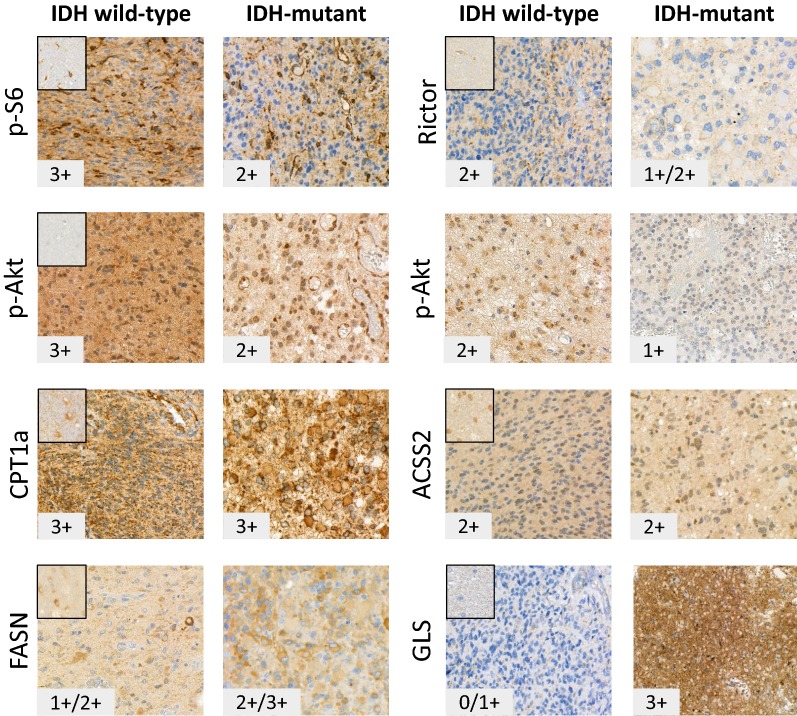
Representative stainings for metabolic protein expression analyses in human glioma biopsies. Metabolic and mTOR pathway related protein expression patterns and evaluated staining scores were shown in representative human IDH wild-type and mutant gliomas. Stainings of normal brain tissues were added to the upper left corner. High mTOR activity—with tissue heterogeneity and characteristic CPT1a protein overexpression (17/18 3+ scores) were detected in almost all studied gliomas. The intra- and intertumoral heterogeneity appeared in e.g. p-S6, FASN and p-(Ser473)-Akt stainings, respectively. Significant differences between IDH wild-type and mutant cases were only observed in GLS expression scores, the IDH-mutant glioma tissues expressed higher amount of GLS protein (expression scores were 2/3+ in 6/8 mutant and 0/1+ in 8/10 studied IDH wild-type cases). Further explanation is in the text. Immunohistochemistry stainings of IDH wild-type (n = 10) and IDH-mutant (n = 8) cases were developed by DAB (brown) using haematoxylin counterstaining—the representative photos were taken by Panoramic Viewer (3D Histech—×40)

Analysing the data of in vitro treatments and comparing these to the protein expression profiles of the cells suggest certain baseline protein expression differences in the studied cell lines. These could correlate to rapamycin + temozolomide effectivity or could help to find which other combinations can result tumour growth inhibition in less sensitive cell lines. In vitro cell lines do not necessarily reflect clinical tumours in all aspects; therefore, metabolic enzyme expressions were studied in human glioma biopsies and peritumoral normal brain tissues by immunohistochemistry, as well. We tested the expressions of certain proteins—which were previously analysed by Western blot—using immunohistochemistry in situ on human biopsy samples (10 wild-type and 8 IDH-mutant cases). Both intratumoral (see in Fig. 7 representative p-S6, p-Akt and FASN staining heterogeneity) and case dependent heterogeneous staining intensities were detected at tissue level. Beside the detected remarkable high mTOR activity—which showed intra- and intertumoral heterogeneity with average score 2+ of all mTOR complex expression and activity markers in the studied cases—high expression of CPT1a was detected in all studied human glioma cases compared to normal brain (high scores—3+—were evaluated in 17/18 studied glioma cases Fig. 7). It was also concluded that ACSS2 and p-(Ser473)-Akt/Rictor expression differences showed high score variability among tumours; IHC staining intensities were scored between 1+ and 3+ in same grade IDH-mutant and wild-type tissues. Additionally, FASN expression was only overexpressed in some cases of the studied biopsy materials. However, GLS overexpression was characteristic for IDH-mutant rather than wild-type gliomas (GLS expression scores were 2/3+ in 6/8 IDH-mutant and 0/1+ in 8/10 studied IDH wild-type cases). The intratumoral heterogeneity in the expression pattern of the studied proteins and the higher anti-GLS staining intensities of IDH-mutant tumour tissues were shown in representative IHC figures (Fig. 7). An implication of these findings—the heterogeneity among the studied IDH wild-type cases—is that the clinical, therapeutic data of glioma cases should be studied in correlation to their in situ metabolic protein expression profiles, especially in case of mTORI and temozolomide treatment resistant patients (e.g. tumour pairs before and after treatments).

## Discussion

Recent standard therapies have certain limitations in terms of the special site in the background of blood–brain barrier, the genetic and tissue heterogeneity and the often developing resistance against different therapies of highly malignant and recurring gliomas [26]. To the present, PI3K/Akt/mTOR signalling beside EGFR is one of the most frequently targeted pathways in different solid tumours and gliomas [26, 27]. Clinical data show that the effects of recently used drugs on GBM patients cannot reach the expectations in stable diseases; e.g. relapses and moreover, on- and off-target effects of these high-dose long-term treatments cannot be tolerated by patients [26]. Dual PI3K/mTOR inhibitors show less toxicity in patients, furthermore, their clinical efficacy is mainly related to genetic alterations e.g. PI3KCA, PTEN mutations which could suggest possible mTORI sensitivity of tumours and could not lower the recurrence. Therefore, more molecular pathology data are needed to find new therapeutic options and these are also needed prior to drug administration in patients. However, many further problems also occur: (a) the great capacity of tumour tissues for shifting their metabolic profile to adapt and survive the therapeutic conditions in the presence of diverse microenvironments [28] and (b) the insufficient cost-benefit in tolerance of therapeutic efficacy and side-effect toxicity in patients (many promising drugs, especially mTOR, glutaminolysis or glycolysis inhibitors have been failed in clinical trials recently).

After applying temozolomide or rapamycin mono-treatments, we found that the studied cells could alter both their glycolytic/OXPHOS and lipid synthetic processes as the consequences of metabolic adaptation. We detected ACSS2 increase and FASN decrease; in parallel, with or without CPT1a expression changes dependent on the cell line examined. These alterations and other recently published data highlight the potential role of lipid and/or acetate utilisation as bioenergetic substrates in survival adaptation during therapy in glioma cells [29]. Our results about the individual differences in FASN and ACSS2 overexpression, and the overexpression of CPT1a in almost all clinical samples suggest the potential role of lipid metabolism in therapy resistance. These and the increased ACSS2 expression after temozolomide or mTORI treatments in vitro correlate to other studies where inhibited glucose consumption triggered AMPK activation and autophagy related acetate consumption in glioma cells [30]. Etomoxir (an irreversible CPT1a inhibitor) can inhibit fatty acid oxidation. Its effect can slow down glioma tumour growth, which was described in mouse glioma models [20] and there are many other data suggesting that fatty acids are critical for GBM growth [28]. In our work, we observed some additional effects of etomoxir combinations, however, these were rather cell type dependent, and less pronounced when this drug was combined with rapamycin or temozolomide. Therefore, the potential importance of etomoxir or other fatty acid oxidation inhibitors need further studies and improvements.

Several successful results have been reported on using temozolomide + autophagy inhibitor combinations [16, 31]. Chloroquine targeting autophagy showed lower sensitivity in the studied cells and different two-drug combinations showed less anti-proliferative benefit with rapamycin, temozolomide or other used drugs. None of these combinations reached the effectivity of rapamycin + temozolomide treatments in the studied glioma cells in vitro. Moreover, combining chloroquine could also reduce the efficiency of two other combined drugs in U373-U cells (data not shown), but could induce the anti-proliferative effect of temozolomide + doxycycline in U87 and rapamycin + doxycycline in U373-U, respectively. These results call the attention to the controversial contribution of autophagy to the development and progression of cancers, and especially in in vivo trials which are more complex [32]. Although in in vivo situation with long-term treatments some unexpected results could also arise with autophagy inhibitors, beside their hopefully special benefits, which need further studies.

Before and after radiotherapy significant differences were described in metabolites, cells rapidly turn to OXPHOS inhibiting mTOR and HK2 in glioma cell line, as a model system [33]. In our work, we detected altered metabolite concentrations but these were the consequences of rather mTORI than temozolomide treatments specific effects. Metabolic enzyme expression changes could also be found after temozolomide treatments in the studied less temozolomide sensitive cell lines (proliferation was decreased by less than 20% after 72 h). Relating to the observed effects of temozolomide suggest some putative shifts towards more OXPHOS-like phenotype in these cells, which is in correlation to the findings of Oliva et al. [34, 35] in case of temozolomide chemoresistance targeted by complex I–IV inhibitors. This work highlighted that mitochondrial metabolism could be a therapeutic avenue for chemoresistance breakthrough.

In our work, we used doxycycline (known mitochondria targeting antibiotics) which was able to sensitise the studied glioma cells to temozolomide. Doxycycline has been tested recently both in vitro and in vivo glioma models, it was very effective in A172 astrocytoma cells [36]. Certain aspects of doxycycline treatment were described by Wang-Gillam et al. in 2007 [37], they detected mild anti-proliferative effect and MMP2 expression increase in high-dose doxycycline (10 µg/mL) in vitro treatment. It was suggested that doxycycline is a potential anti-migratory agent in TSC2 dysfunction (mutation) cases—but this combination was not further tested elsewhere [38]. The anti-tumoural properties of doxycycline and other antibiotics are intensively investigated by several groups to clear the effect of this drug on tumour stemness, as well [39]. According to our work, the synergistic anti-proliferative effects of doxycycline in combination with other drugs are suggested. We studied doxycycline + rapamycin treatment first in glioma cell proliferation and we found that doxycycline enhanced the anti-proliferative effect of rapamycin in all studied glioma cells.

These results suggest the importance of testing the effectivity and the other—such as pro-apoptotic or tissue environmental—effects of this combination further in vitro and in vivo in gliomas or other tumours (we have some promising results with other solid tumour cell lines, as well—data not shown). Additionally, rapalogs or antibiotics cause fewer side-effects for patients and can be combined with other tumour type specific reduced dose chemotherapeutic treatments.

The detected diversity in the metabolic changes of the studied glioma cell lines could not point out a clear correlation between the original metabolic phenotype and the subsequent expected metabolic alteration in response to certain treatments. To find such potential adaptation mechanisms related to original phenotype, tumour type and/or drug responses need further investigation. Based on our in vitro metabolic drug combination results, we can suggest using combined treatments against different metabolic pathways which will restrict the possibilities of adaptation and rescue mechanisms in many different tumour cells during the developing therapy resistance.

Based on our IHC results: (a) the expression of many metabolic enzymes showed intra- and intertumoral heterogeneity; (b) CPT1a expression is elevated in human glioma cases (our results confirm the potential importance of CPT1a expression in gliomas which was described in vitro by Wakamiya et al. [40]); and (c) GLS is overexpressed in IDH-mutant cases (the importance of glutamine metabolism was highlighted as special interest in glioma cases by others, as well [41]). These and the results of our in vitro metabolic drug combination treatments suggest considering the use of more potential metabolic targets in glioma therapy and analysing these targets further using more clinical samples and survival data of patients. These could help to identify new markers beside mTOR activity characterisation—such as FASN, CPT1a, ACSS2—or find good mitochondrial function markers (e.g. TOM20, NRF1), and to assign the metabolic expression profile of tumours before starting treatments in patients. mTOR inhibitors alone or in combinations with traditional chemotherapeutics resulted mainly at below the expected breakthrough. The main causes could be the tissue heterogeneity, the metabolic plasticity and the generated alterations in treated cells. The dormant, resistant state can also correlate to phenotypic OXPHOS shifts and the mitochondrial functions. Our results could approve this possibility in glioma cells. In addition, we call the attention for new rapalog + doxycycline therapeutic combinations in the future with potentially fewer side-effects.

Targeting altered mitochondrial function to increase sensitivity was suggested previously to inhibit complex I in gliomas using other drugs like metformin or phenformin [42]. These drugs show promising anti-tumour effects in multiple cancers which were tested and confirmed in glioma in vitro and in vivo xenograft models [43]. Based on our data, doxycycline needs some more attention in GBM treatment because it has long and safe history; furthermore, it can also penetrate across the blood–brain barrier [44]. Additionally, doxycycline could be considered as a potential efficacious combining agent among the few available ones in GBM therapy, especially with rapamycin or other mTORI treatments. Moreover, rapamycin + doxycycline combination could have a special interest in the standard treatment of other therapy resistant tumours, as well.

## Conclusion

Metabolic heterogeneity and plasticity attract the attention to tumour metabolism targeting as an anti-cancer therapy, however, tumour tissue is able to adapt more successfully to nutrient deprivation or other microenvironmental changes than (normal) cells in vitro. To predict these alterations further studies are needed in tumour metabolism which might lead to select prognostic and predictive markers. Nevertheless, certain already well-known agents combined with tumour metabolism targeting drugs could facilitate the success of recent tumour therapy. Based on these, there is a great hope that new or old metabolic inhibitors such as mTOR inhibitors and other metabolism targeting drugs will turn the recent therapy highly effective in patients, especially in gliomas.

## Abbreviations

4EBP1: eukaryotic translation initiation factor 4E (eIF4E)-binding protein 1
ACSL1: long-chain-fatty-acid-CoA ligase 1
ACSS2: acyl-coenzyme A synthetase short-chain family member 2
AGC: protein kinase A, G, and C families
AKG: α-ketoglutarate
Akt: protein kinase B
AMPK: AMP-activated protein kinase
CDKN2A/B: cyclin-dependent kinase inhibitor 2A/B
CPT1a: carnitine palmitoyltransferase 1A
CST: Cell Signaling Technology
DAB: 3,3′-diaminobenzidine
DMSO: dimethyl sulfoxide
ECL: enhanced chemiluminescence
EGFR: epidermal growth factor receptor
FASN: fatty acid synthase
FBS: foetal bovine serum
FFPE: formalin-fixed, paraffin-embedded
GBM: glioblastoma multiforme
GLS: glutaminase
HK2: hexokinase 2
HRP: horseradish peroxidase
IDH: isocitrate dehydrogenase
IHC: immunohistochemistry
LC-MS: liquid chromatography-mass spectrometry
LDHA: lactate dehydrogenase A
LDHB: lactate dehydrogenase B
MMP2: matrix metalloproteinase-2
MRM: multiple reaction monitoring
MSH2: MutS protein homolog 2
mTOR: mammalian target of rapamycin
NF-1: neurofibromatosis 1
Notch-2: neurogenic locus notch homolog protein 2
NRF1: nuclear respiratory factor 1
OXPHOS: oxidative phosphorylation
PDGFRA: platelet-derived growth factor receptor A
PDH: pyruvate dehydrogenase
PFKP: phosphofructokinase
PI3K: phosphatidylinositol 3-kinase
PI3KCA: phosphatidylinositol 3-kinase catalytic subunit A
PKC: protein kinase C
PTEN: phosphatase and tensin homolog
PVDF: polyvinylidene difluoride
S6K1: ribosomal protein S6 kinase beta-1
SGK: serum/glucocorticoid-regulated kinase
SRB: sulforhodamine B
SREBP1: sterol regulatory element-binding protein 1
TCA: tricarboxylic acid
TMA: tissue microarray
TOM20: translocase of outer membrane
TSC2: tuberous sclerosis complex 2
ULK1: Unc-51 like autophagy activating kinase 1
VEGF: vascular endothelial growth factor
β-F1-ATPase: β-F1-adenosine triphosphate synthase
